# Supplementary material for: CA1 pyramidal cell diversity is rooted in the time of neurogenesis
Source: eLife. 2021 Nov 1;10:e69270. doi: 10.7554/eLife.69270 (PMC8660020; doi:10.7554/eLife.69270)
Supplement: Transparent reporting form [file elife-69270-transrepform.docx]

***eLife’s* transparent reporting form**

We encourage authors to provide detailed information *within their submission* to facilitate the interpretation and replication of experiments. Authors can upload supporting documentation to indicate the use of appropriate reporting guidelines for health-related research (see [EQUATOR Network](http://www.equator-network.org/%20)), life science research (see the [BioSharing Information Resource](https://biosharing.org/)), or the [ARRIVE guidelines](http://www.plosbiology.org/article/info:doi/10.1371/journal.pbio.1000412) for reporting work involving animal research. Where applicable, authors should refer to any relevant reporting standards documents in this form.

If you have any questions, please consult our Journal Policies and/or contact us: [editorial@elifesciences.org](mailto:editorial@elifesciences.org).

**Sample-size estimation**

- You should state whether an appropriate sample size was computed when the study was being designed
- You should state the statistical method of sample size computation and any required assumptions
- If no explicit power analysis was used, you should describe how you decided what sample (replicate) size (number) to use

Please outline where this information can be found within the submission (e.g., sections or figure legends), or explain why this information doesn’t apply to your submission:

We did not compute the sample size when the study was designed because it is impossible to know exactly how many neurons will be fate-mapped a priori with our method due to the variability of tamoxifen-induced and Cre-mediated recombination in embryos. That said, sample-size (i.e. number of mice, of slices, of neurons) was considered to be large enough to apply proper statistical testing and as it is clearly indicated throughout the manuscript as detailed below.

**Replicates**

- You should report how often each experiment was performed
- You should include a definition of biological versus technical replication
- The data obtained should be provided and sufficient information should be provided to indicate the number of independent biological and/or technical replicates
- If you encountered any outliers, you should describe how these were handled
- Criteria for exclusion/inclusion of data should be clearly stated
- High-throughput sequence data should be uploaded before submission, with a private link for reviewers provided (these are available from both GEO and ArrayExpress)

Please outline where this information can be found within the submission (e.g., sections or figure legends), or explain why this information doesn’t apply to your submission:

We have reported throughout the manuscript, in the results, methods and figure legends, how often each experiment was performed. Number of recorded neurons ex vivo, of neurobiotin-filled cells for morphology as well as exclusion criteria are given in the methods section. Outlier values were excluded from ex vivo electrophysiological recordings as indicated in the methods sections (ROUT method, Q = 0.1%, GraphPad Prism). Number of neurons included for the analysis of radial cell distribution, putative PV innervation, CB immunostaining, cFos analysis and retrograde tracing experiments are given in the results section and/or in the source data files. Detailed information about samples used to generate the results for all experiments is included as source data files to each figure.

**Statistical reporting**

- Statistical analysis methods should be described and justified
- Raw data should be presented in figures whenever informative to do so (typically when N per group is less than 10)
- For each experiment, you should identify the statistical tests used, exact values of N, definitions of center, methods of multiple test correction, and dispersion and precision measures (e.g., mean, median, SD, SEM, confidence intervals; and, for the major substantive results, a measure of effect size (e.g., Pearson's r, Cohen's d)
- Report exact p-values wherever possible alongside the summary statistics and 95% confidence intervals. These should be reported for all key questions and not only when the p-value is less than 0.05.

Please outline where this information can be found within the submission (e.g., sections or figure legends), or explain why this information doesn’t apply to your submission:

The submission includes a recapitulative excel file with detailed information on all statistical tests and methodology used for each of experiment. When relevant, p-values and confidence intervals are clearly indicated.

(For large datasets, or papers with a very large number of statistical tests, you may upload a single table file with tests, Ns, etc., with reference to sections in the manuscript.)

**Group allocation**

- Indicate how samples were allocated into experimental groups (in the case of clinical studies, please specify allocation to treatment method); if randomization was used, please also state if restricted randomization was applied
- Indicate if masking was used during group allocation, data collection and/or data analysis

Please outline where this information can be found within the submission (e.g., sections or figure legends), or explain why this information doesn’t apply to your submission:

Samples were allocated into different experimental groups depending on the age of tamoxifen administration or on the age of the mice.

**Additional data files (“source data”)**

- We encourage you to upload relevant additional data files, such as numerical data that are represented as a graph in a figure, or as a summary table
- Where provided, these should be in the most useful format, and they can be uploaded as “Source data” files linked to a main figure or table
- Include model definition files including the full list of parameters used
- Include code used for data analysis (e.g., R, MatLab)
- Avoid stating that data files are “available upon request”

Please indicate the figures or tables for which source data files have been provided:

We have uploaded, with this submission, source data files linked to all the figures and tables. We also have uploaded (on a Dryad account associated to the submission) raw files and code (if applicable).
